# Physicochemical Characterisation of KEIF—The Intrinsically Disordered N-Terminal Region of Magnesium Transporter A

**DOI:** 10.3390/biom10040623

**Published:** 2020-04-17

**Authors:** Stéphanie Jephthah, Linda K. Månsson, Domagoj Belić, Jens Preben Morth, Marie Skepö

**Affiliations:** 1 Division of Theoretical Chemistry, Department of Chemistry, Lund University, Naturvetarvägen 14, 221 00 Lund, Sweden; stephanie.jephthah@teokem.lu.se (S.J.); linda.mansson@live.com (L.K.M.); 2 Division of Physical Chemistry, Department of Chemistry, Lund University, Naturvetarvägen 14, 221 00 Lund, Sweden; domagoj.belic@fkem1.lu.se; 3 Enzyme and Protein Chemistry, Section for Protein Chemistry and Enzyme Technology, Department of Biotechnology and Biomedicine, Technical University of Denmark, Søltofts Plads, 2800 Kgs. Lyngby, Denmark; premo@dtu.dk; 4 Lund Institute of Advanced Neutron and X-ray Science (LINXS), Scheelevägen 19, 233 70 Lund, Sweden

**Keywords:** membrane proteins, intrinsically disordered proteins, circular dichroism spectroscopy, small-angle X-ray scattering, cryogenic transmission electron microscopy, molecular-dynamics simulations, protein–vesicle interactions, magnesium transporter, secondary structure

## Abstract

Magnesium transporter A (MgtA) is an active transporter responsible for importing magnesium ions into the cytoplasm of prokaryotic cells. This study focuses on the peptide corresponding to the intrinsically disordered N-terminal region of MgtA, referred to as KEIF. Primary-structure and bioinformatic analyses were performed, followed by studies of the undisturbed single chain using a combination of techniques including small-angle X-ray scattering, circular dichroism spectroscopy, and atomistic molecular-dynamics simulations. Moreover, interactions with large unilamellar vesicles were investigated by using dynamic light scattering, laser Doppler velocimetry, cryogenic transmission electron microscopy, and circular dichroism spectroscopy. KEIF was confirmed to be intrinsically disordered in aqueous solution, although extended and containing little *β*-structure and possibly PPII structure. An increase of helical content was observed in organic solvent, and a similar effect was also seen in aqueous solution containing anionic vesicles. Interactions of cationic KEIF with anionic vesicles led to the hypothesis that KEIF adsorbs to the vesicle surface through electrostatic and entropic driving forces. Considering this, there is a possibility that the biological role of KEIF is to anchor MgtA in the cell membrane, although further investigation is needed to confirm this hypothesis.

## 1. Introduction

In a society where antimicrobial resistance is constantly manifesting in new ways, the demand for effective antibiotics is naturally increasing. In order to rationalise the design of new antibiotics, and to find new potential cellular targets, bacterial biochemical functions must be mapped and fully understood.

The magnesium ion Mg^2+^ is the most abundant divalent cation in any biological system and, it being an essential mineral nutrient and thus an absolute requirement for life, is present in every cell type in every living organism [1]. In cells, Mg^2+^ is an essential cofactor for more than 600 enzymes, including important DNA and RNA polymerases; it is also required for stabilisation of the ribosome–protein complex during protein synthesis. In adenosine triphosphate (ATP)-dependent enzymes, Mg^2+^ binds an ATP, the main unit of cellular energy, in the catalytic pocket, thus activating the phosphate ester towards hydrolysis [2]. In non-ATP-dependent enzymes, the role of Mg^2+^ is instead to hold a water molecule in a specific position, and this water molecule in turn helps to hold a particular structure in place or participates directly in the enzymatic reaction mechanism [2].

In bacteria and archaea, three major classes of Mg^2+^ transporters have been identified as responsible for the translocation of Mg^2+^ across the cell membrane: channel protein CorA, gated channel protein magnesium transporter E (MgtE), and pump magnesium transporters A and B (MgtA and MgtB) [2]. Only MgtA and MgtB serve as primary active transporters, but unlike other P-type ATPases, MgtA and MgtB mediate Mg^2+^ influx down, rather than against, the electrochemical gradient. Mg^2+^ acts as a product inhibitor for MgtA, which is activated by free Mg^2+^ concentrations below 10 μM and strongly inhibited by concentrations above 1 mM [3].

MgtA consists of 898 amino acids and has a molecular weight of 99.5 kDa. In a recent study by Subramani et al., (2016) [3], using the DISOPRED3 server for intrinsically disordered region (IDR) prediction [4], the first 33 amino acids (1–33) of MgtA from *Escherichia coli (E. coli)* were classified as intrinsically disordered. How the disordered nature of this IDR affects the biological function of MgtA however remains unknown. Thus, the focus of this study is to investigate this N-terminal, intrinsically disordered part of MgtA, hereafter referred to as KEIF. KEIF has the following amino acid sequence:
M F KE I F T R L I R H L P S R L V H RD P L P G A Q Q T V N T V.
At physiological pH, KEIF carries five positively charged (blue) and two negatively charged (red) amino acids, giving it a net charge of +3. The majority of charged amino acids are evenly distributed in the first half of the peptide.

Here, we present the first-ever physicochemical characterisation of KEIF, which was performed using a variety of computational and experimental techniques. First, primary-structure and bioinformatic analyses were performed in order to make predictions about the overall structure and behaviour of the peptide. Second, experimental techniques such as circular-dichroism (CD) spectroscopy and small-angle X-ray scattering (SAXS) were used in combination with atomistic molecular-dynamics (MD) simulations to characterise the undisturbed single chain in aqueous solution. Interactions with neutral and anionic large unilamellar vesicles (LUVs) were investigated by using dynamic light scattering (DLS), laser Doppler velocimetry (LDV), cryogenic transmission electron microscopy (cryo-TEM), and CD spectroscopy. Corroborating Subramani’s DISOPRED3-based prediction [3], we found KEIF to display typical characteristics of a disordered peptide in aqueous bulk solution. Interestingly, dissolution in an organic solvent or the presence of anionic vesicles serve both to induce an increase of helical structure within the peptide. The obtained results might help shine some light on KEIF’s role for the function of MgtA, as the question still remains: does KEIF play an important role in MgtA function, or should KEIF be regarded merely as a passive appendix?

## 2. Materials and Methods

### 2.1. Sample Preparation

#### 2.1.1. Samples for CD Spectroscopy

KEIF powder (95.67%; Genemed Synthesis Inc., San Antonio, TX, USA) was dissolved in, and was purified by dialysis (100–500 Da MWCO Biotech Cellulose Ester (CE) Dialysis Membrane Tubing; SpectrumLabs, Piraeus, Greece) against, a 20 nM tris(hydroxymethyl)aminomethane (TRIS) buffer at pH 7.4 and at 6 °C. Following purification, the concentration of the peptide stock solution was determined using a Thermo Scientific NanoDrop 2000 Spectrophotometer (Waltham, MA, USA) (UV-Vis at 214 nm, *ε*/1000 = 60.805 $M^{‒1}$ ${\mathrm{cm}}^{‒1}$, *Mw* = 3.871 kDa). The stock solution was diluted with additional buffer to prepare 0.2 mg mL^−1^ (52 μM) samples for CD measurements, supplemented with either 10 or 150 mM NaF, and sometimes with 1 mM (corresponding to ∼20 eq.) CaCl_2_, MgCl_2_ or ZnCl_2_. Samples were filtered (0.22 μm MILLEX-GV Filter Unit) prior to measurements.

To prepare a sample of KEIF in 2,2,2-trifluoroethanol (TFE), KEIF powder was first dissolved in, and was purified by dialysis (100–500 Da MWCO Biotech Cellulose Ester (CE) Dialysis Membrane Tubing; SpectrumLabs, Piraeus, Greece) against, milliQ water at 6 °C. The sample was then lyophilised and the resulting purified powder was dissolved in TFE (>99%; Sigma-Aldrich, Stockholm, Sweden) to yield 0.2 mg mL^−1^ (52 μM). This sample was not filtered prior to measurements as filter units do not withstand organic solvents.

#### 2.1.2. SAXS Samples

A 20 mM TRIS buffer was prepared and acidified with HCl to maintain a pH of 7.5. The ionic strength of the buffer was set to 140 mM using NaCl. KEIF powder was dissolved in the buffer and dialysed against the same buffer at 4 °C, using a 500–1000 Da MWCO Regenerated Cellulose (RC) Dialysis Membrane Tubing (SpectrumLabs, Piraeus, Greece). Before SAXS measurements, samples were centrifuged at 18,400 RCF at 6 °C for at least 30 min to remove impurities and aggregates. Protein concentrations were measured immediately before SAXS measurements using a NanoDrop One Microvolume UV-Vis Spectrophotometer (UV-Vis at 214 nm).

#### 2.1.3. LUV Samples

**Figure 1:**
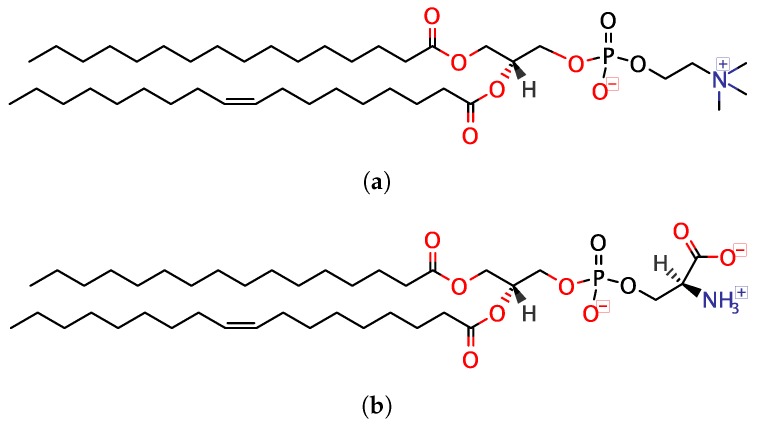
Lipids used for preparation of large unilamellar vesicles (LUVs): (**a**) 1-palmitoyl-2-oleoyl-glycero-3-phosphocholine (POPC) and (**b**) 1-palmitoyl-2-oleoyl-sn-glycero-3- phospho-L-serine (POPS).

Neutral LUVs were prepared using 1-palmitoyl-2-oleoyl-glycero-3-phosphocholine (POPC; Avanti Polar Lipids, Alabaster, AL, USA), whereas anionic ones were prepared using a 3:1 (mol:mol) mixture of POPC and 1-palmitoyl-2-oleoyl-sn-glycero-3-phospho-L-serine (POPS; Avanti Polar Lipids, Alabaster, AL, USA) (Figure 1). Lipids were (co)dissolved in 3:7 (v:v) methanol:chloroform in a glass vial. Solvents were evaporated under a stream of nitrogen, after which the resulting lipid film was further dried under reduced pressure (0.8 bar) overnight. The lipids were then hydrated with 20 mM TRIS buffer at pH 7.4 to a total lipid concentration of 30 mM, and the sample was then subjected to five freeze–thaw cycles before being extruded 31 times through a 0.1 μm polycarbonate membrane filter (Avanti Polar Lipids, Alabaster, AL, USA).

#### 2.1.4. LUV–KEIF Samples

KEIF (purified by dialysis as described in Section 2.1.1) was mixed with POPC or 3:1 POPC:POPS LUVs to give final peptide and lipid concentrations of 750 μM and 12.0 mM, respectively. The used buffer was 20 mM TRIS at pH 7.4, and ionic strength was set to 10 mM with NaF. Samples were incubated overnight before DLS (Section 2.2.3) and LDV measurements (Section 2.2.4), and CD spectroscopy (Section 2.2.1). Cryo-TEM imaging (Section 2.2.5) was performed the following day. Samples were used as for cryo-TEM imaging, but were diluted six times (to 125 μM peptide and 2.0 mM lipid) prior to CD measurements, and five more times (to 25 μM peptide and 0.4 mM lipid) prior to DLS and LDV measurements.

### 2.2. Experiment Methods

#### 2.2.1. CD Spectroscopy

Far-UV CD spectra were recorded in 0.1 nm intervals (typically) between 190 and 260 nm, with four accumulations, on a JASCO J-715 spectropolarimeter equipped with a photomultiplier tube detector. Samples were measured in a 1 mm quartz cuvette (Hellma Analytics 110-QS). Temperature control was ensured using a PTC-348WI peltier-type temperature-control system. The measurement temperature was 20 °C, and samples were equilibrated for 5 min at this temperature prior to measurements. All spectra were corrected by subtracting a reference spectrum obtained from a sample lacking KEIF, but which was otherwise identical. Ellipticity is reported as mean residue molar ellipticity θ (degcm^2^dmol^‒1^) according to Equation (1), where $\theta_{obs}$ is ellipticity (deg), mrw is mean residue molecular weight, *c* is protein concentration (g mL^−1^), and *l* is the optical path length of the cell (cm).

(1) $$\theta =\theta_{obs}\left(mrw\right)/10lc$$

Some of the obtained CD spectra were subject to BeStSel [5,6] fitting through a web-server (http://bestsel.elte.hu/index.php) to access the corresponding secondary structure elements. Fitted residuals are presented in Figures S1 and S2.

#### 2.2.2. SAXS Experiments

SAXS experiments were performed at beamline BM29 at the European Synchrotron Radiation Facility (ESRF) in Grenoble, France. The incident-beam wavelength was 0.99 Å, and the distance between sample and PILATUS 1M detector was set to 2869 mm. The temperature of the storage and exposure cells was 20 °C. By measuring the scattering of pure water, the forward scattering I(0) was converted to an absolute scale. At least ten successive frames with an exposure time of 1 s were recorded for each sample. Scattering from the pure solvent was also measured both before and after each individual protein-sample measurement, and subtracted from the corresponding protein-sample spectrum. Special attention was paid to radiation damage by comparing successive frames prior to background subtraction in order to avoid inclusion of faulty data. Data were processed and analysed using the ATSAS package [7]. The ensemble optimisation method (EOM) [8,9] was used to fit theoretical scattering intensities to the experiment data.

#### 2.2.3. DLS Measurements

A Malvern Zetasizer Nano-ZS (Malvern Instruments Ltd., Malvern, UK) equipped with a 633 nm 4 mW HeNe laser with automatic laser attenuator was used for DLS measurements. Disposable PMMA cuvettes (BRAND GMBH, Wertheim, Germany) were used as sample cells. The measurement temperature was 20 °C and samples were equilibrated for 5 min prior to measurements. Measurements were performed at a fixed scattering angle of 173° using the noninvasive backscatter (NIBS) technique. Data were analysed by the cumulant method provided by the instrument software. Hydrodynamic radius $R_{H}$ (Z-average radius, or “cumulant mean”, given by the software) is given as the average of five consecutive measurements of 60 s where standard deviation represents the error.

#### 2.2.4. LDV Measurements

A Malvern Zetasizer Nano-ZS (Malvern Instruments Ltd., Malvern, UK) equipped with a 633 nm 4 mW HeNe laser with an automatic laser attenuator was used for LDV measurements to obtain estimates of electrophoretic mobility. Disposable folded capillary cells (Malvern DTS1070) were used as sample cells. The measurement temperature was 20 °C, and samples were equilibrated for 5 min prior to measurements. Measurements were performed at a fixed scattering angle of 17° using the M3-PALS laser interferometric technique. Electrophoretic mobility is given as the average of three consecutive measurements where standard deviation represents the error.

#### 2.2.5. Cryo-TEM Imaging

Cryo-TEM sample preparation and imaging were performed at the National Center for High-Resolution Electron Microscopy within Lund University. Lacey formvar-carbon film on 200 mesh copper TEM grids (Ted Pella, Redding, CA, USA) were glow-discharged in a Quorum GloCube system (Quorum Technologies, Laughton, UK). Then, 4 μL of vesicle suspension was pipetted onto the TEM grid in a Leica EM GP automatic plunge freezer (Leica Microsystems, Stockholm, Sweden) operating at 21 °C and relative humidity of >90%, backside-blotted for 2.5 s, and plunged into liquid ethane. Samples were transferred onto a Fischione 2550 cryogenic sample holder and imaged on a JEOL JEM-2200FS (JEOL, Tokyo, Japan) transmission electron microscope equipped with an omega energy filter, operating at an accelerating voltage of 200 kV. Sample temperature was kept below −174 °C during imaging. The zero-loss images were acquired on an F416.0 camera (TVIPS, Gauting, Germany) using Serial EM software [10] running in low-dose mode (total electron dose per image < 15 e^−^ Å^−2^). The acquired cryo-TEM images were processed using ImageJ software [11].

### 2.3. Calculations

#### 2.3.1. Isoelectric-Point Calculation

Theoretical isoelectric-point (pI) calculation was performed using the Swiss Institute of Bioinformatics (SIB) Bioinformatics Resource Portal ExPASy (Expert Protein Analysis System) [12] Compute pI/Mw tool through a web-server (https://web.expasy.org/compute_pi/).

#### 2.3.2. Partitioning-Free-Energy Calculation

Water-to-bilayer partitioning-free-energy calculation was performed using the Membrane Protein Explorer (MPEx) tool [13] and the Wimley–White octanol scale [14,15]. His was considered neutral, Lys and Arg positive, Asp and Glu negative, and both termini charged.

### 2.4. Simulations

#### 2.4.1. Atomistic MD Simulations

Atomistic MD simulations were performed using the GROMACS package (version 4.6.7) [16,17,18], with an AMBER ff99SB-ILDN force field [19] and TIP4P-D water model [20]. A rhombic dodecahedron was used as a simulation box, with periodic boundary conditions in all directions. A minimal distance of 1 nm was set between solute and box edges. An initial, linear structure of KEIF was built using PyMOL [21]. The two His residues in the amino acid sequence were set to be neutral throughout the simulations, giving the peptide a net charge of +3. Three chloride ions were added to neutralise the system. Simulations were performed without the addition of any salt.

The equations of motion were integrated using the Verlet leap-frog algorithm [22] with a time step of 2 fs. A Verlet list cut-off scheme was used for the nonbonded interactions. Short-ranged interactions were calculated using a pair list with a cut-off of 1 nm. Long-ranged dispersion interactions were applied to the systems’ energy and pressure, and long-ranged electrostatics was managed by using Particle Mesh Ewald [23] with cubic interpolation and a grid spacing of 0.16 nm. All bond lengths were constrained using the LINCS algorithm [24]. A velocity-rescaling thermostat [25] with a relaxation time of 0.1 ps was used to keep a temperature of 300 K. A Parrinello–Rahman pressure coupling [26] was used to keep pressure constant at 1 bar throughout the simulations. Relaxation time was 2 ps, and isothermal compressibility was set to that of water, i.e., $4.5\times 10^{-5}$ bar^−1^.

Energy minimisation was done using the steepest-descent algorithm. Initiation was performed in two steps with position restraints on the peptide to equilibrate the temperature and pressure of the systems: (1) 500 ps NVT simulations, and (2) 1000 ps NPT simulations. Five replicates with different starting seeds were used for each simulation. The production runs were also performed in the NPT ensemble, and were run for a total of 10 μs (5 × 2 μs).

#### 2.4.2. Simulation Analyses

The average radius of gyration ($R_{g}$) and end-to-end distance ($R_{ee}$) were obtained using the GROMACS tool g_polystat. To assess the convergence of the simulations, autocorrelation functions and block-error estimates of $R_{g}$ and $R_{ee}$ were computed using the GROMACS tool g_analyze. Principal component analysis (PCA) of the peptide backbone, by Campos and Baptista [27], and by using only the first two PCs, was also used for the evaluation of convergence and sampling. The minimal distance between periodic images in the simulations was monitored by the GROMACS tool g_mindist to ensure that the simulated peptide did not interact with its periodic images. Cluster analysis was done with the GROMACS tool g_cluster, using the GROMOS method [28], and was also used to obtain frames for representative structures. All peptide structures were visualised and rendered using PyMOL [21]. Distance matrices were obtained by using the GROMACS tool g_mdmat, and were used to create distance maps to show the distance between amino acid residues within the peptide. To create a contact map for the entire trajectory, MDTraj research software was used [29]. The secondary structure was analysed using the GROMACS tool g_rama and the DSSP program (version 2.2.1) [30], as well as DSSPPII analysis, that is, the DSSP program with modifications by Chebrek et al. [31] to include detection of the polyproline II (PPII) helix. Theoretical scattering intensities were obtained by using CRYSOL (version 2.8.2) [32].

## 3. Results and Discussion

The physicochemical characterisation of KEIF was performed in three parts by using a variety of methods. First, the primary structure was analysed to predict the overall structure and behaviour of the peptide. This was done by evaluating the charge, disorder propensity, and hydrophobicity/hydrophilicity per amino acid residue in the KEIF sequence. In addition, the sequence was compared with other protein sequences and sequence motifs from various protein databases. Second, the single chain was studied using far-UV CD spectroscopy, SAXS, and atomistic MD simulations. These results yielded basic structural properties, such as the average radius of gyration, maximal dimension ($D_{\max}$), and end-to-end distance, as well as distance-distribution functions (P(r)) and secondary-structure information. Third, interactions with neutral and anionic LUVs were investigated using DLS, LDV, cryo-TEM, and CD spectroscopy.

### 3.1. Primary-Structure Analysis

#### 3.1.1. Charge-Distribution, Isoelectric-Point, and Das–Pappu Analysis

**Figure 2:**
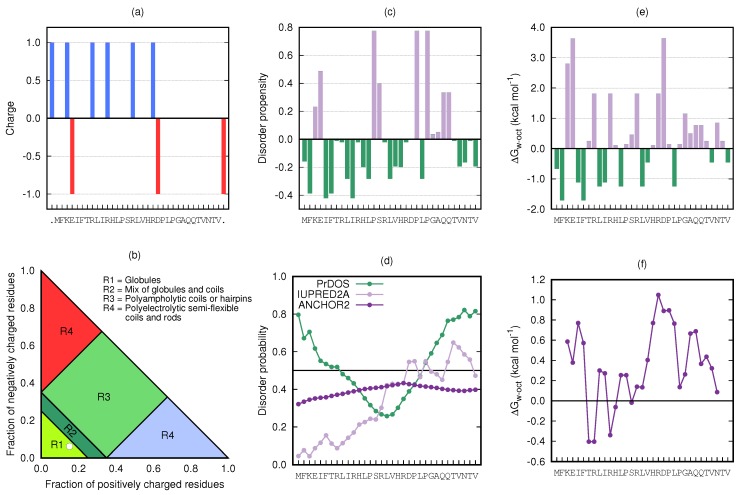
Primary structure analyses of KEIF: (**a**) Estimated charge per amino acid residue. Charge of peptide termini included as separate residues and marked as dots in the x-axis label. (**b**) Das–Pappu plot [33,34]. KEIF location is indicated by white circle in Region R1. (**c**) Disorder propensity per amino acid $\left(C_{\mathrm{DisProt}}-C_{\mathrm{PDB}}\right)/C_{\mathrm{PDB}}$, as described by Uversky (2013) [35]. (**d**) Probability prediction of disordered regions and disordered binding regions using PrDOS (green) [36], IUPred2A (light purple), and ANCHOR2 (dark purple) algorithms [37]. (**e**) Whole-residue Wimley–White hydrophobicity indices [14,15] per amino acid residue. (**f**) Kyte–Doolittle [38] smoothed (five amino acid sliding-window) hydropathy plot based on whole-residue Wimley–White indices.

The estimated charge per amino acid at pH 7.0 is shown in Figure 2a, where the majority of the charged residues were located in the N-terminal half of the sequence. At this pH, the contribution from the histidines (pK_a_ 6.0) to the total charge was assumed to be negligible, giving a peptide net charge of (about) +3. The isoelectric point of KEIF was calculated to be 11.54 using the ExPASy [12] tool Compute pI/Mw. KEIF was predicted to belong to the R1 region of the Das–Pappu plot [33,34] in Figure 2b, which predicted that KEIF assumes a globular structure in aqueous solution.

#### 3.1.2. Disorder Propensity and Probability

Figure 2c shows the disorder propensity per amino acid based on fractional difference $\left(C_{\mathrm{DisProt}}-C_{\mathrm{PDB}}\right)/C_{\mathrm{PDB}}$ as described by Uversky (2013) [35]. Overall, the sequence did not seem to contain a substantial amount of disorder-promoting residues, although a cluster of disorder-promoting residues was found closer to the C-terminal end of the sequence (residues Pro-24–Gln-28), suggesting that this part of the peptide has higher propensity for disordered conformations. The obtained prediction by the IUPred algorithm [37] in Figure 2d also suggested a low probability of disorder that increased sightly towards the C-terminus. This observation is, however, not supported by PrDOS analysis [36], which instead predicted disorder at both termini, and only a low probability of disorder in the central part of the sequence. The probability of disordered binding regions (by ANCHOR2 [37]) was found to be low across the entire amino acid sequence.

#### 3.1.3. Distribution of Hydrophobic and Hydrophilic Amino Acids

The distribution of hydrophobic and hydrophilic amino acid residues in KEIF is depicted in Figure 2e, where the whole-residue Wimley–White hydrophobicity indices [14,15]—corresponding to the free energy ΔG of transfer from water to *n*-octanol—were taken as a measure of amino acid hydrophobicity/hydrophilicity. As revealed by the hydropathy plot shown in Figure 2f, obtained from Kyte–Doolittle sliding-window analysis [38], the peptide was overall (slightly) hydrophilic in character, and no transmembrane regions could be identified; with a typical bilayer thickness of 30 Å, an α-helical transmembrane segment would have to involve approximately twenty amino acids, and a β-strand nine. The hydrophilic character returned from analysis suggested that KEIF does not reside in the transmembrane part of MgtA, but likely protrudes into the surrounding aqueous environment either intracellularly or extracellularly.

#### 3.1.4. Sequence and Motif Alignment

The amino acid sequence of KEIF was compared with sequences from other proteins in the UniProtKB/Swiss-Prot database [39] with a protein-similarity search [40,41,42,43] to see if there were any similar sequences with known function. Pairwise sequence alignment of the top six results is displayed in Table 1. Excluding MgtA sequences, neither one of the resulting sequences had a high score, and the expected values indicated no biological significance. In addition, all matching sequences were found well within their corresponding proteins, which made any similarities of function unlikely. Smaller fragments of the charged part of the sequence (residues 3–21) were investigated in the same way, but yielded no different results. The full KEIF sequence was tested for containing any sequence motifs using ScanProsite [44] and MOTIF [45], but none was found.

### 3.2. Single Chain

#### 3.2.1. CD Spectroscopy

**Figure 3:**
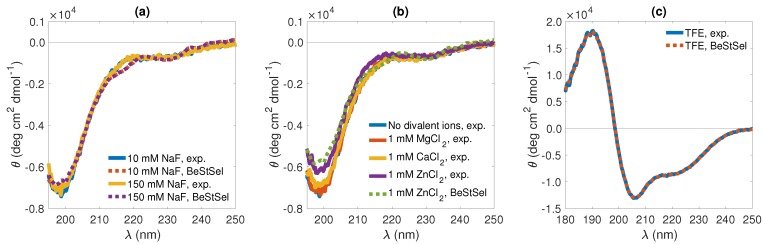
KEIF circular-dichroism (CD) spectra (solid lines) with BeStSel [5,6] fits (dashed lines), showing the effect of (**a**) varying salt (NaF) concentration (in TRIS buffer), (**b**) introducing various divalent cations (10 mM NaF in TRIS buffer), and (**c**) switching to organic solvent (TFE).

CD spectroscopy, a technique widely used to study the conformation of proteins in solution [46,47,48], was used with KEIF in order to obtain information about the peptide’s secondary structure. CD spectra were recorded at 10 and 150 mM 1:1 salt (NaF), on the addition of Mg^2+^, Ca^2+^ and Zn^2+^ cations in the form of chloride salts, as well as in organic solvent TFE (Figure 3a, Figure 3b and Figure 3c, respectively). In aqueous solution (TRIS buffer) and irrespective of salt concentration, the obtained CD spectra were characteristic of a disordered structure [46,47], and appeared to be completely insensitive to a 15-fold change in salt concentration (Figure 3a). The disordered structure is likely promoted by intrachain electrostatic repulsion caused by the relatively high density of positively charged amino acid residues. As expected, on the basis of the high similarity of the two spectra, the BeStSel [5,6] fitting of the two datasets returned highly similar secondary-structure elements where irregular (other) structures constituted the largest portion (see Table 2). The fits also pointed to a considerable fraction of β-strands, whereas helical structure elements were absent.

Whereas KEIF secondary structure appeared to be essentially insensitive to the presence of divalent Ca^2+^ and Mg^2+^ cations, again deduced from recorded CD spectra, the addition of Zn^2+^ ions served to make the minimum at around 200 nm somewhat less pronounced (Figure 3b); however, the effect on the corresponding structural elements returned from BeStSel [5,6] fitting is almost negligible. KEIF’s apparent insensitivity to the presence of divalent cations was not surprising, considering that amino acids typically involved in metal ion co-ordination via their polar side-chain atoms—thiolate-carrying Cys (C), imidazole-carrying His (H), and carboxylate-carrying Glu (E) and Asp (D), collectively known as CHED [49]—are scarce. Moreover, the rather high density of cationic amino acid residues, as opposed to anionic ones, likely makes KEIF–cation interactions electrostatically unfavourable.

The situation was very different when KEIF is suspended in TFE (Figure 3c). In this organic solvent, as indicated by the development of a double minimum at 208 and 220 nm and a maximum at 192 nm [46,47], helical content considerably increases, mainly at the expense of the portion of β-strands (Table 2). Similar observations were made for the human-saliva protein histatin 5, which has disordered conformation in aqueous solution [50], but adopts a more helical conformation in TFE [51,52].

#### 3.2.2. SAXS Measurements

**Figure 4:**
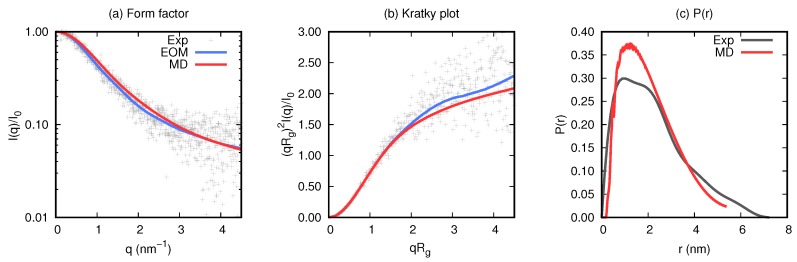
Experiment small-angle X-ray scattering (SAXS) results (grey) compared to ensemble-optimisation-method (EOM; blue) and molecular-dynamics (MD) simulations (red). (**a**) Form factors, (**b**) Kratky plot, and (**c**) distance-distribution functions.

Conformational information about the single chain of KEIF was obtained by performing SAXS experiments. The resulting form factor, Kratky plot, and distance-distribution function are depicted in Figure 4, in comparison to the EOM fit and obtained results from MD simulations. Figure 4a shows the obtained form factor, whose shape indicated natively unfolded behaviour. Further investigation of the data, in the form of the Kratky plot (Figure 4b), revealed the typical curve shape of a fully flexible and extended protein/peptide. The EOM fit conformed well with the experiment data ($\chi^{2}=1.143$).

Estimations of the radius of gyration were obtained using Guinier approximation (up to $qR_{g}\leq 0.8$), the P(r) and the EOM. As shown in Table 3, Guinier approximation provides the smallest estimation, and P(r) the largest, although the difference between the two was only 0.1 nm (5.5%). The estimation from the EOM was close to an average of the two values, and corresponded to deviations of only 2.2–3.3%. Estimations of the maximal dimension were also obtained from the P(r) and the EOM, which are also shown in Table 3. A larger discrepancy of approximately 2 nm (33.3%) was found between the two estimated values.

#### 3.2.3. Atomistic Simulations

Atomistic MD simulations were performed to complement the experiment studies, and to obtain additional insight about the conformational properties of KEIF in bulk solution. Simulation convergence was assessed considering probability-distribution functions, autocorrelation functions, and block-average-error estimates of the radius of gyration and end-to-end distance (see Figures S3–S6). PCA was also utilised for this assessment (Figure S7). Discussion of the convergence is referred to the Supplementary Materials. To assess the validity of the simulations, simulation results were compared to the experiment results. Scattering curves were procured from the concatenated simulation trajectory by the use of CRYSOL (version 2.8.2) [32] and compared to the experiment SAXS curves and the curves from the EOM (see Figure 4). The curves were found to be very similar. The radius of gyration from the simulation was, however, found to be smaller than what was obtained from analysis of the experiment data (see Table 3), although the percentage difference was only 7.1–12.6%. Because of the good correspondence with the experiment SAXS results, the simulated data were considered to be sufficiently valid to be used as accurate single-chain representation.

**Figure 5:**
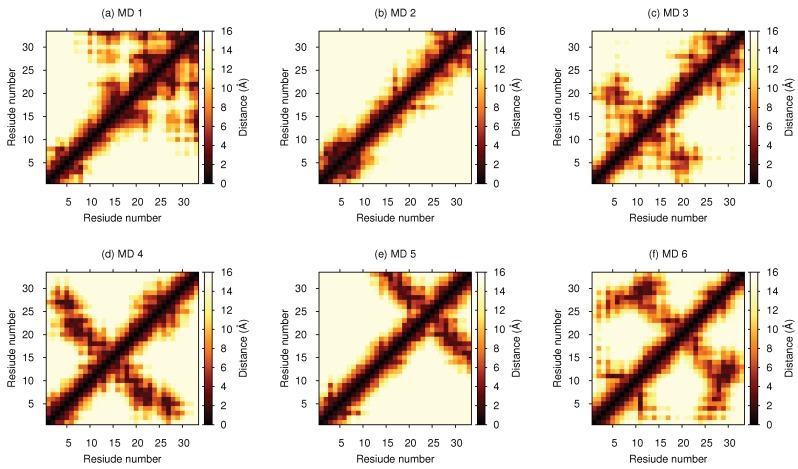
Distance maps depicting distance between amino acid residues for each representative structure of top six clusters from MD simulation.

**Figure 6:**
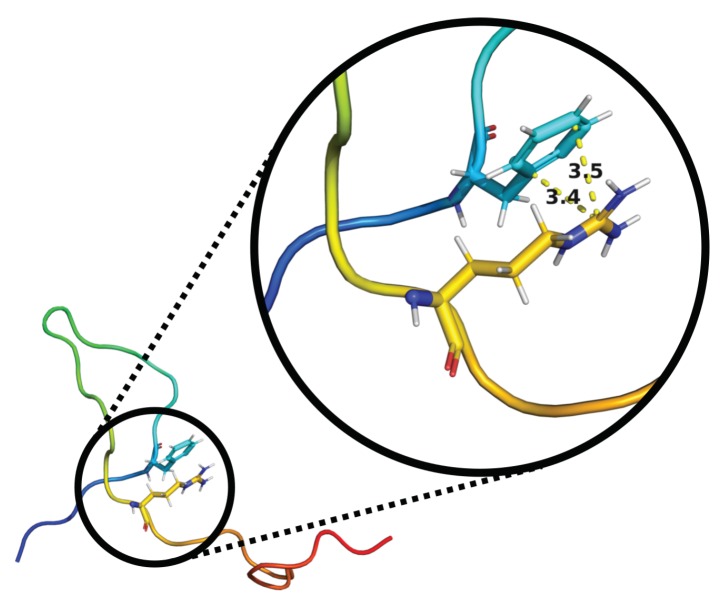
Illustration of cation–π interaction between Phe-6 and Arg-20 in MD 3 structure. marked distances given in Ångström (Å).

**Figure 7:**
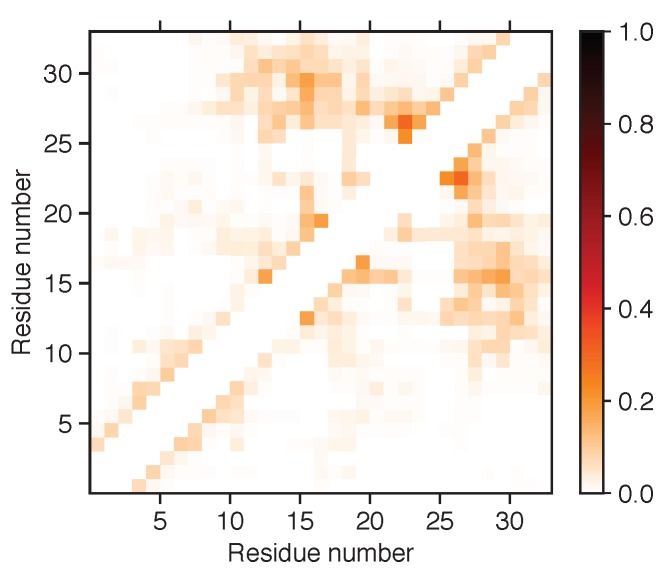
Contact map showing the probability of amino acid residues being closer to each other than cutoff of 4.0 Å. Darker colour indicates higher probability, and white corresponds to zero probability. Residue interactions with themselves, as well as two neighbouring residues on each side, were excluded from analysis.

Cluster analysis was performed on the concatenated MD simulation trajectory to obtain representative structures. Eight clusters were found with an RMSD cutoff of 0.99 Å, and the top six (99.75%) were compared to the six structures that were obtained from EOM analysis in Table 4. A large majority of the MD structures were found in the first two clusters at this cutoff. However, if using an RMSD cutoff of 0.70 Å or 0.50 Å, cluster sizes became of more equal size, and the top eight clusters summed up to 58.72% and 16.74%, respectively. For more thorough analysis of the structures, distance maps showing the distance between amino acid residues in the representative structures were created (see Figure 5). By studying these maps, details otherwise unnoticed were found. For example, evidence of cation–π interactions was observed between Phe-6 and (i) Arg-20 in the MD 3 structure (see Figure 6), (ii) Gln-27 in the MD 4 structure, and (iii) Lys-3 in the MD 5 structure. The remaining close distances seemed to arise due to hydrogen bonds and dispersion interactions, although a few electrostatic interactions were also observed. A contact map, instead showing the probability of contacts within a cutoff of 4.0 Å throughout the entire concatenated simulation, is presented in Figure 7. Here, the most probable contact was found between Leu-23 and Gln-27. Other notable contacts were found between residues Leu-13 and Arg-16, Arg-16 and Val-30, as well as between Leu-17 and Arg-20.

**Figure 8:**
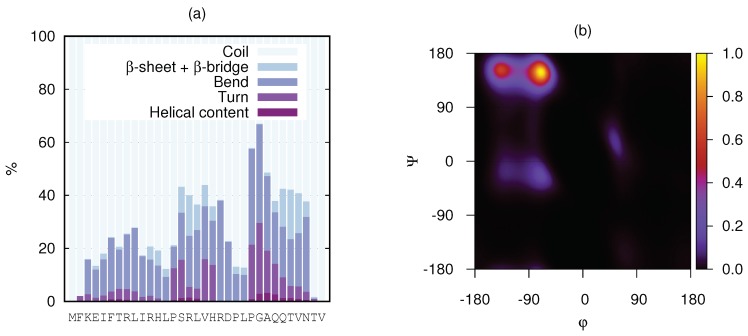
(**a**) Stacked secondary-structure histograms per amino acid residue of KEIF as obtained from MD simulations. Three different types of helices included in helical content: (i) α-helix, (ii) $3_{10}$-helix, and (iii) π-helix. This analysis did not include the PPII helix. (**b**) Ramachandran plot of KEIF as obtained from MD simulations.

The secondary structure per amino acid of KEIF from the MD simulation was analysed using the DSSP algorithm, and is visualised in Figure 8a. Although most of the structure was dominated by coils and bends, a few residues also showed propensity for turns and β-structures. The helical content was found to be negligible. Unfortunately, this analysis did not include the PPII helix. To account for PPII helices, DSSPPII analysis was utilised on the representative structures from the top six clusters of the MD simulations (see Table 5). While the N-terminal half of the first structure was dominated by random coil conformation, a more local order was found towards the C-terminus as distinguished turns around a small PPII helix at Asp-21–Pro-22–Leu-23, followed by an isolated β-bridge between Thr-29 and Thr-32. The small PPII helix around residues 21–24 was conserved in the top three cluster structures, although PPII helices were present in all structures. Particularly, the fourth structure seemed to have strong PPII propensity. A small $3_{10}$-helix was found at residues 4–6 in the second structure, whereas the sixth structure contained evidence of β-sheet formation. These results did not contradict what was observed by CD spectroscopy (Figure 3). The presence of PPII in the conformational ensemble of KEIF was also in line with what was seen in the Kratky plot (Figure 4), that is, mainly flexible but extended conformations. A Ramachandran plot (Figure 8b) was also produced from the simulated results that showed a high count in the region of $\left(\phi ,\psi \right)=\left(-75^{\circ },+145^{\circ }\right)$, which also supported a significant PPII content. The plot also shows a fairly high count of β-structures, but only little α-helical content, which corroborated the CD spectroscopy results (Table 2).

### 3.3. Interactions with Vesicles

The interaction of KEIF with lipid bilayers, provided by neutral (POPC) and anionic (POPC:POPS) LUVs, was characterised by partitioning-free-energy calculations, DLS, LDV, cryo-TEM, and CD spectroscopy, as outlined in the following subsections. The anionic bilayer may be regarded as a model of the bacterial-cell membrane, while the neutral one was used for comparison to elucidate the effect of membrane charge and the importance of electrostatic interactions.

#### 3.3.1. Partitioning Free Energies

The hydrophilic/hydrophobic character of a peptide or a protein naturally influences its interactions with a bilayer. In Section 3.1.3 it was concluded that KEIF has an overall (slightly) hydrophilic character, and should consequently prefer bulk water rather than the hydrophobic interior of a bilayer. To support this hypothesis, the MPEx tool [13] was used to calculate the free energy ΔG of partitioning of KEIF from bulk water into a bilayer. By applying the MPEx tool to KEIF, water-to-bilayer partitioning free energy of +18.21 kcal mol^‒1^ was obtained, signifying the unfavourableness of water-to-bilayer partitioning. Even if partitioning would present an opportunity to reduce the free energy by a partitioning–folding coupling mechanism—corresponding to approximately 0.4 kcal mol^‒1^ per amino acid residue [53]—free energy would still be positive (+5.01 kcal mol^‒1^), and water-to-bilayer partitioning thus unflavoured. KEIF–vesicle interactions discussed in the following subsections are instead attributed to electrostatic interactions between net positive KEIF and net negative POPC:POPS vesicles.

#### 3.3.2. DLS and LDV

Zwitterionic lipid POPC (carrying one positively charged and one negatively charged functional group; net charge ±0) and anionic lipid POPS (carrying one positively charged and two negatively charged functional groups; net charge −1) were used for the preparation of LUVs. The vesicles were prepared by extrusion, resulting in monomodal size distributions (polydispersity index, PdI<0.1) with diameters *D* centred at 108.0 nm and 102.9 nm for the neutral POPC and anionic 3:1 POPC:POPS vesicles, respectively, as measured by DLS (correlation functions are shown in Figure S8). LDV, in turn, was used to determine vesicle electrophoretic mobility, μ. Whereas a considerable net negative mobility was measured for the POPC:POPS vesicles due to the anionic nature of POPS, the mobility of the POPC vesicles could not be accurately determined due to their extremely weak net charge; the value given for the POPC vesicle mobility has to thus be taken with a grain of salt. Vesicle-size and electrophoretic-mobility data are summarised in Table 6.

Initially, KEIF-vesicle interactions were probed by by DLS. DLS is highly sensitive to changes in particle size, as scattered intensity *I* scales with the sixth power of particle radius *r* ($I\propto r^{6}$). The day after KEIF addition (∼18 h), vesicle-size distributions were still monomodal (correlation functions are shown in Figure S8), reflecting the absence of vesicle aggregation. Whereas the size of neutral POPC vesicles slightly increased upon addition of KEIF (Table 6)—possibly an indication of fusion of a small number of vesicles—the size of anionic 3:1 POPC:POPS ones was, instead, somewhat reduced. We hypothesise that cationic KEIF electrostatically adsorbs to the surface of the anionic vesicles to neutralise some of the negative charges, thereby reducing the lateral head-group repulsion and allowing lipids to pack closer.

Second, electrophoretic-mobility measurements were performed to study the adsorption of KEIF to vesicle surface. Whereas the mobility of the neutral POPC vesicles became only somewhat more positive upon addition of positively charged KEIF (remember, however, that these low mobility values are not too accurate), the mobility of the anionic 3:1 POPC:POPS vesicles became significantly more positive (Table 6). On the basis of these measurements, we hypothesise that KEIF, only to a (very) small extent, adsorbs to the POPC vesicles; KEIF can possibly access the negative charge on a small number of the POPC head groups. The apparent high affinity of KEIF for the anionic POPC:POPS vesicles signifies that a net-negative vesicle charge is important for adsorption, with charge neutralisation and concomitant entropy gain resulting from counter ion release being the main driving force for adsorption. The importance of charges and electrostatic interactions was also reported elsewhere [54]. Moreover, the fact that mobility—a measure of surface charge—becomes significantly more positive upon KEIF addition to the anionic 3:1 POPC:POPS vesicles supports the hypothesis that KEIF adsorbs to the vesicle surface and does not partition into the hydrophobic interior of the lipid bilayer. That into-bilayer partitioning is unfavourable stems from the hydrophilic nature of KEIF (Figure 2f), which is reflected in the positive partitioning free energies presented in Section 3.3.1.

#### 3.3.3. Cryo-TEM

**Figure 9:**
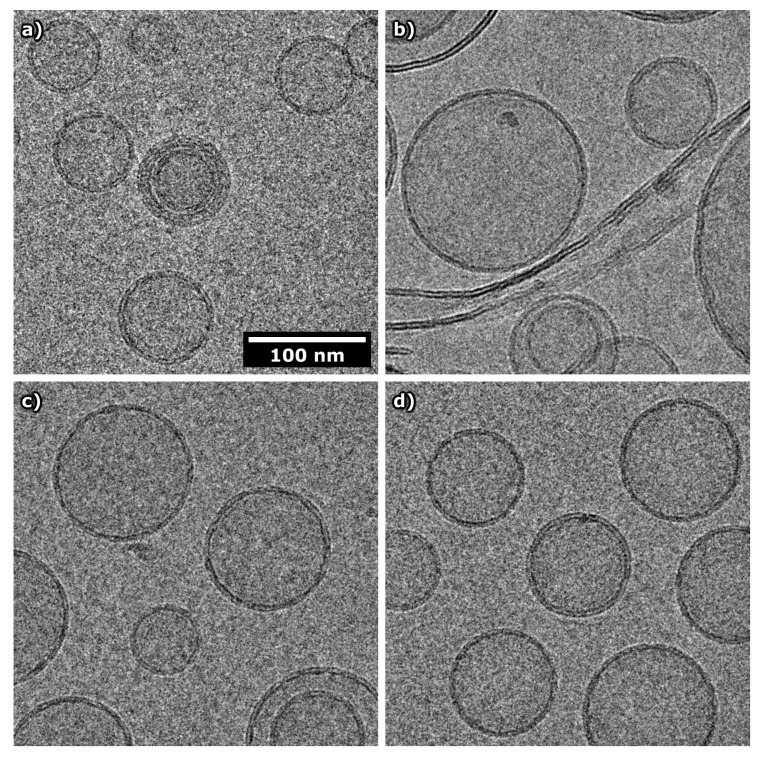
Representative cryo-TEM images of (**a**,**b**) POPC and (**c**,**d**) 3:1 POPC:POPS vesicles in the (**a**,**c**) absence and (**b**,**d**) presence of KEIF, at 10 mM NaF in TRIS buffer. The lipid:KEIF molar ratio was 16:1. The scale bar applies to all images.

In order to obtain complementary information to that provided by DLS, POPC and 3:1 POPC:POPS vesicles were imaged by cryo-TEM [55], in the absence and presence of KEIF (Figure 9; additional images are shown in Figures S9 and S10). Whereas the majority of the anionic 3:1 POPC:POPC vesicles were unilamellar, multilamellar vesicles were frequently observed in the case of the neutral POPC vesicles. Vesicles with one or two smaller-sized internalised vesicles were observed in both cases, but were more common with POPC. In both cases, the degree of polydispersity appeared to be larger than that indicated by DLS (Table 6), which was supported by the larger standard deviation returned from cryo-TEM image analysis of vesicle size (Figure S11).

The addition of KEIF to the neutral POPC vesicles had a dramatic effect on vesicle stability, as evidenced by a severe degree of polydispersity—likely caused by fusion of the original, small-sized vesicles—and the presence of a large number of ruptured vesicles and free lipid bilayers. This state did not alter the macroscopic appearance of the sample, nor was it picked up by the DLS measurements, which still indicated a low polydispersity index (Table 6). We hypothesise that extremely weakly charged POPC vesicles, on addition of KEIF polyelectrolytes, become electrostatically destabilised, and thus prone to rupture and fusion. In contrast to POPC vesicles, the anionic 3:1 POPC:POPS vesicles seemed to be unaffected by the addition of KEIF, as these vesicles remained intact. Corroborating the observations made by DLS, a smaller size was measured for the POPC:POPS vesicles in the presence of KEIF (Figure S11). As previously discussed, adsorption of KEIF to the vesicles likely reduces lipid head-group repulsion, thus enabling lipids to pack closer.

#### 3.3.4. CD Spectroscopy

CD spectroscopy is a useful tool for monitoring conformational changes in membrane-active proteins and peptides. In such studies, as model membranes, small unilamellar vesicles (SUVs) made by sonication have been used almost exclusively, as the common belief has been that light scattering associated with LUVs is unacceptably high. It was shown, however, that undistorted CD spectra can be obtained at wavelengths above 200 nm in the presence of up to 3 mM LUVs [56]. By choosing LUVs over SUVs, vesicle-curvature effects that may cause anomalous peptide partitioning are reduced, and, as LUVs are thermodynamically stable structures (as different from SUVs which are only metastable), equilibrium thermodynamic measurements can be performed.

**Figure 10:**
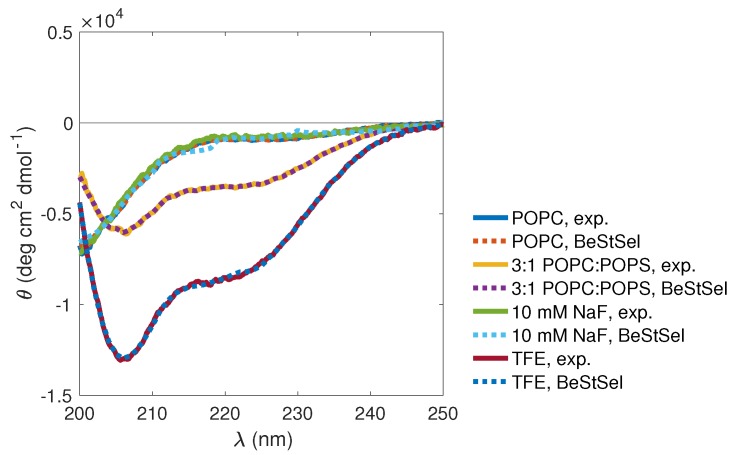
KEIF CD spectra recorded in presence of POPC and 3:1 POPC:POPS vesicles, in TRIS buffer supplemented with 10 mM NaF. Lipid:KEIF molar ratio was 16:1. For comparison, spectra recorded in TRIS buffer supplemented with 10 mM NaF and in TFE are also shown. Dashed lines represent BeStSel [5,6] fits.

Using CD spectroscopy, the possible induction of secondary-structure elements in KEIF upon adsorption onto the aforementioned neutral POPC and anionic 3:1 POPC:POPS LUVs was studied. Obtained CD spectra, recorded the day after KEIF addition to the vesicles, showed clear differences between POPC and POPC:POPS vesicles (Figure 10). First, the spectrum recorded in the presence of neutral POPC vesicles was highly similar to that recorded in the absence of vesicles, and BeStSel [5,6] fitting returned similar amounts of various secondary structural elements (Table 7). This means that either adsorption to the POPC vesicles does not induce any conformational changes or, more likely and supported by obtained results form DLS and LDV (Section 3.3.2), KEIF simply does not posses any electrostatic affinity for the POPC vesicles and consequently does not adsorb.

The spectrum recorded in the presence of anionic POPC:POPS vesicles (Figure 10) is clearly different from that recorded in the presence of neutral POPC vesicles (or in the absence of vesicles), which again highlights the importance of electrostatic interactions to drive the adsorption of KEIF to the vesicles. Changes to the CD spectrum induced by the POPC:POPS vesicles suggest an increase in the secondary-structure content of KEIF, from a largely disordered conformation in solution to more ordered conformation when absorbed to the vesicles. The spectrum showed the same characteristic features as the spectrum recorded in TFE, with a double minimum at 208 and 220 nm that is indicative of helical structure [46,47]. Indeed, BeStSel [5,6] fitting of the spectrum showed an increase in helical content (see Table 7).

### 3.4. Summary of, and Correlations between, Main Results

Primary-structure analysis predicted a rather globular conformation of KEIF, with larger disorder propensity towards the C-terminus of the peptide. No disordered binding regions were predicted, nor were any sequence motifs or transmembrane regions found. Both CD and SAXS experiments showed that KEIF is indeed a disordered peptide in aqueous solution, in agreement with Subramani’s DISOPRED3-based prediction [3]. These results were also supported by MD simulations, for which the scattering curve was in good agreement with experiment SAXS data (Figure 4). Contrary to predictions, SAXS results suggested that KEIF is fully flexible and extended instead of globular. This was also supported by the simulations, where the radius of gyration was found to be similar (within 7.1–12.6%; Table 3), and the largest conformation clusters were represented by fairly extended structures (Table 4). Thus, solely relying on net charge and the fraction of charged residues for predicting the conformation and shape of the peptide seemed to be insufficient in this particular case, and implied that the location of the charges in the sequence is of significance. With all of the positive charges evenly distributed in the N-terminal half of the sequence, it is not unexpected that electrostatic repulsion could cause the extension of this part of the peptide chain.

Analysis of the CD spectroscopy results suggested a little amount of β-structures in the single chain in aqueous solution (Table 2), which was also observed in the DSSP analysis and in the Ramachandran plot of the MD simulations (Figure 8). By performing DSSPPII analysis on the simulated peptide, PPII structure was also revealed to be an important secondary-structure component, mainly found towards the C-terminal end of the peptide (Table 5). This discovery cannot be disregarded by the experiment results, since the PPII helix was not included in theBeStSel analysis. Furthermore, CD spectroscopy of KEIF in organic solvent TFE induced a considerable increase of helical content.

Partitioning-free-energy analysis deemed water-to-bilayer partitioning of KEIF to be unfavourable. DLS and LDV results seemed to agree with this prediction, and provided data suggesting that KEIF instead has the ability to adsorb to the surface of anionic POPC:POPS vesicles (Table 6). In addition, CD spectroscopy revealed a conformational change for KEIF with this type of membrane, inducing an increase in helical content (Figure 10 and Table 7), similarly to what was observed with KEIF suspended in TFE, but to a smaller extent. We hypothesise that these observations are attributed to the electrostatic adsorption of cationic KEIF to the anionic vesicle surface, driven by charge neutralisation and a concomitant release of counterions. This would also explain the apparent low propensity of KEIF to adsorb to neutral POPC vesicles.

## 4. Conclusions

This paper featured the extensive physicochemical characterisation of KEIF, the N-terminal disordered region of MgtA, using an approach combining various experimental techniques and MD simulations. Both the experimental techniques and the complementary simulations confirmed that KEIF is an extended intrinsically disordered peptide with little propensity towards β-structures, and possibly PPII structure. In addition, experiments showed that KEIF adopts a more helical structure in organic solvent TFE. Further experimental investigation of interactions between KEIF and vesicles proved that it is unlikely for KEIF to traverse the bilayer, and that it instead seems to adsorb to the surface of anionic vesicles. Because of opposite charges of the vesicles and the peptide, it is hypothesised that the interaction is electrostatically driven. KEIF adsorption to the vesicle surface also implies a release of counterions, which is entropically favourable and constitutes an additional driving force for adsorption.

**Figure 11:**
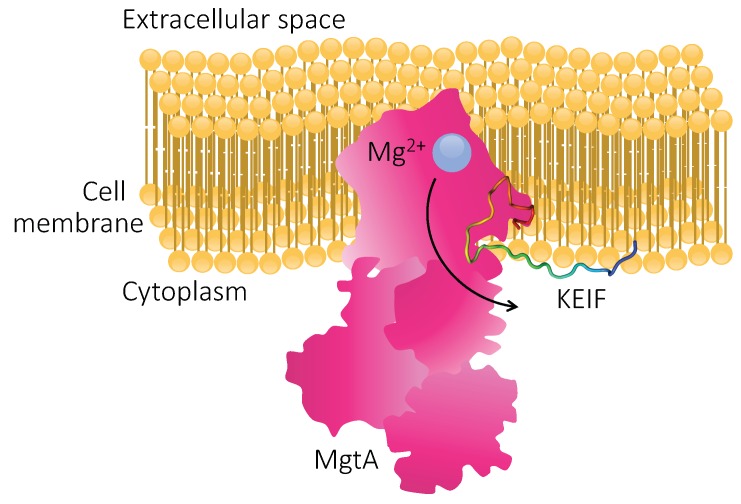
Schematic cross-section of an MgtA-carrying cell membrane. It is possible that KEIF plays the role of an anchor, helping to stabilise the large protein complex in the membrane by locking it in place via electrostatic interactions with anionic lipid head groups.

By performing this study, we provided comprehensive insight to the structure–function relationship of KEIF that, in turn, might aid in providing a more holistic understanding of the function of MgtA. Because of the observed interactions between KEIF and anionic lipid bilayers, it is reasonable to believe that this intrinsically disordered region of MgtA actually has an important function in the biological context. Considering KEIF’s hydrophilic character, together with its electrostatic affinity for the surface of anionic lipid bilayers (the bacterial cell membrane being a prime example), its role might be to anchor the large MgtA protein in the bilayer, as schematically depicted in Figure 11. Whereas additional research, beyond the scope of this paper, is required in order to elucidate the exact role of KEIF, it does seem likely that KEIF is more than just a mere “appendix” of MgtA.

## Figures and Tables

**Table 1:** Pairwise sequence alignment, score, and expect value for top six results obtained from amino acid sequence similarity search of KEIF.

	Start Res. No.	Sequence	Stop Res. No.	Score (Bits)	E-Value
KEIF	1	MFKEIFTRLIRHLPSRLVHRDPLPGAQQTVNTV	33	-	-
Query	1	MFKEIFTRLIRHLPSRLVHRDPLPGAQQTVNTV	33		
		MFKEIFTRLIRHLPSRLVHRDPLPGAQQTVNTV		70.5	1 × 10${}^{-15}$
Sbjct 1	1	MFKEIFTRLIRHLPSRLVHRDPLPGAQQTVNTV	33		
Query	3	KEIFTRLIRHLPSRLVHRDPLPGAQQTVN	31		
		+++F RL RHLP RLVHRDPLPGAQ VN		48.5	6 × 10^−8^
Sbjct 2	7	RQLFARLNRHLPYRLVHRDPLPGAQTAVN	35		
Query	2	FKEIFTRLIRHLPSRLVHRDPLPGAQQTVNTV	33		
		FKE+ +L+ L ++HR+P P Q N V		28.5	0.8
Sbjct 3	785	FKEVEVQLLPELEEMILHRNPFPALQTLRNRV	816		
Query	2	FKEIFTRLIRHLPSRLVHRD	21		
		F+E+ T + RHLP L H+D		26.9	3.0
Sbjct 4	178	FEEVDTNVTRHLPHELQHKD	197		
Query	2	FKEIFTRLIRHLPSRLVHRDPLPGAQQTVNTV	33		
		FKE+ +L+ L ++HR+P P Q N V		26.2	5.6
Sbjct 5	785	FKEVEVQLLPELEEMILHRNPFPALQTLRNRV	816		
Query	7	TRLIRHLPSRLVHRDPLPG	25		
		TR++RH +R + R+P PG		25.8	7.7
Sbjct 6	129	TRILRHAMTRHIFREPAPG	147		

Sbjct 1 = P0ABB8 Magnesium-transporting ATPase, P-type 1 *(Escherichia coli)*; Sbjct 2 = P36640 Magnesium- transporting ATPase, P-type 1 *(Salmonella typhimurium)*; Sbjct 3 = Q14667 Protein KIAA0100 *(Homo sapiens)*; Sbjct 4 = Q758B8 GPI ethanolamine phosphate transferase 2 *(Ashbya gossypii)*; Sbjct 5 = Q5SYL3 Protein KIAA0100 *(Mus musculus)*; Sbjct 6 = C9K7C0 O-methyltransferase AMT9 *(t)*.

**Table 2:** Estimated secondary structure content in KEIF, returned from BeStSel [5,6] fitting of CD spectra in Figure 3.

	10 mM NaF (aq.)	150 mM NaF (aq.)	1 mM ZnCl_2_ (aq.)	TFE (org.)
**Fitted Range (nm)**	**190–250**	**190–250**	**190–250**	**180–250**
Helix (%)	0.0	0.0	0.0	30.3
β-strand (%)	38.5	38.8	41.5	10.4
Turn (%)	14.9	14.9	14.8	15.9
Others * (%)	46.6	46.3	43.7	43.4

* 3_10_-helix, π-helix, bends, β-bridge, and irregular/loop.

**Table 3:** Ensemble averages of $R_{g}$ and $D_{\max}$ (if applicable) as obtained from various methods.

	$R_{g}$ (nm)	$D_{\max}$ (nm)
Guinier	1.76 ± 0.11	-
P(r) *	1.86	7.22
EOM *	1.80	5.16
MD	1.64 ± 0.05	-

* No explicit errors were given using these analysis methods.

**Table 4:** Comparisons between representative structures from cluster analysis of MD simulations (red) and structures obtained from EOM analysis of experiment SAXS data (blue). The percentage of all structures that belonged to each cluster is given in the parentheses; MD clusters summed up to 99.75%, and EOM structures summed up to ∼100%. RMSD values (Å) of aligned atoms given below each comparison.

	EOM 1 (∼30%)	EOM 2 (∼30%)	EOM 3 (∼10%)	EOM 4 (∼10%)	EOM 5 (∼10%)	EOM 6 (∼10%)
**MD 1** (61.72%)	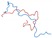	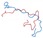	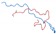	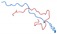	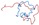	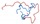
	12.84	10.88	11.32	12.92	10.58	10.45
**MD 2** (24.44%)	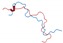	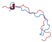	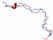	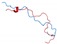	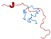	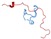
	7.15	7.80	5.81	7.95	16.36	16.33
**MD 3** (8.35%)	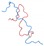	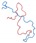	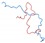	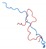	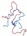	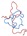
	11.56	12.76	13.45	15.00	8.74	9.25
**MD 4** (3.05%)	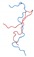	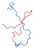	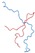	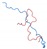	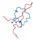	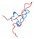
	15.98	16.62	16.69	19.10	10.09	10.28
**MD 5** (1.40%)	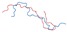	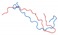	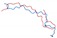	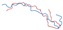	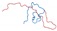	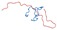
	9.19	7.61	6.44	7.26	15.96	16.26
**MD 6** (0.80%)	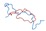	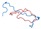	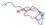	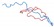	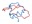	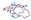
	16.57	15.78	17.16	18.21	5.92	7.28

**Table 5:** Secondary structure per amino acid of representative structures from top six clusters of MD simulation of KEIF, as obtained by using DSSPPII analysis [31]. Secondary structure per amino acid represented according to standard DSSP classification.

#	MFKEIFTRLIRHLPSRLVHRDPLPGAQQTVNTV
1	----SS----S----TTTS-PPPTTTT-BTTB-
2	---GGGTSPP--------S--SPP-S--SS---
3	-----------SS---SPP-PPPTT--SS----
4	---PPPBPP----TTS-----B-TTTTPPPP--
5	------PP------SS--B-PP-TT--SB----
6	----PP-SS-EE-PPPP--SS-----SSEE---

**Table 6:** Z-average diameter, polydispersity index, and electrophoretic mobility of POPC and 3:1 POPC:POPS vesicles in absence and presence of KEIF, at 10 mM NaF in TRIS buffer. Lipid:KEIF molar ratio was 16:1.

	*D* (nm)	PdI	*μ* (10^−8^m^2^V^−1^s^−1^)
POPC vesicles	108.0 ± 0.7	0.09 ± 0.02	−0.12 ± 0.03 *
POPC vesicles + KEIF	111.1 ± 0.6	0.06 ± 0.01	+0.16 ± 0.03 *
3:1 POPC:POPS vesicles	102.9 ± 0.7	0.06 ± 0.02	−3.99 ± 0.61
3:1 POPC:POPS vesicles + KEIF	95.7 ± 0.3	0.07 ± 0.02	−2.02 ± 0.40

* These values should be taken with a grain of salt, as measurement accuracy was low due to the very weak charge.

**Table 7:** Estimated secondary-structure content in KEIF, returned from BeStSel [5,6] fitting of CD spectra in Figure 10.

	POPC (aq.)	3:1 POPC:POPS (aq.)	10 mM NaF (aq.)	TFE (org.)
**Fitted Range (nm)**	**200–250**	**200–250**	**200–250**	**200–250**
Helix (%)	1.8	9.9	0	20.5
β-strand (%)	31.3	27.6	31.0	16.6
Turn (%)	17.7	16.0	17.7	15.2
Others * (%)	49.4	46.5	51.3	47.7

* 3_10_-helix, π-helix, bends, β-bridge, and irregular/loop.

## Supplementary Materials

The following are available online at https://www.mdpi.com/2218-273X/10/4/623/s1. Figure S1: Residuals from BeStSel fitting of CD spectra 1; Figure S2: Residuals from BeStSel fitting of CD spectra 2; Figure S3: MD probability-distribution functions; Figure S4: MD autocorrelation functions; Figure S5: MD block-average-error estimates; Figure S6: MD convergence; Figure S7: PCA free-energy landscapes; Figure S8: DLS autocorrelation functions; Figure S9: Cryo-TEM images 1; Figure S10: Cryo-TEM images 2; Figure S11: Cryo-TEM size distributions.
