# Supplementary material for: Physicochemical Characterisation of KEIF—The Intrinsically Disordered N-Terminal Region of Magnesium Transporter A
Source: Biomolecules. 2020 Apr 17;10(4):623. doi: 10.3390/biom10040623 (PMC7226168; doi:10.3390/biom10040623)
Supplement: Supplementary file 1 [file biomolecules-10-00623-s001.pdf]

Supplementary Materials: Physicochemical Characterisation of KEIF – The Intrinsically Disordered N-terminal Region of Magnesium Transporter A

Stéphanie Jephthah [1], Linda K. Månsson [1], Domagoj Belić [2], Jens Preben Morth [3] and Marie Skepö [1,4,*]

[1] Division of Theoretical Chemistry, Department of Chemistry, Lund University, Naturvetarvägen 14, 221 00 Lund, Sweden
[2] Division of Physical Chemistry, Department of Chemistry, Lund University, Naturvetarvägen 14, 221 00 Lund, Sweden
[3] Enzyme and Protein Chemistry, Section for Protein Chemistry and Enzyme Technology, Department of Biotechnology and Biomedicine, Technical University of Denmark, Søltofts Plads, 2800, Kgs. Lyngby, Denmark
[4] Lund Institute of Advanced Neutron and X-ray Science (LINXS), Scheelevägen 19, 233 70, Lund, Sweden
* Correspondence: marie.skepo@teokem.lu.se; Tel.: +46-46-222 33 66

1. Residuals from BeStSel fitting of CD spectra

Some of the obtained CD spectra were subject to BeStSel [1,2] fitting to access KEIF's corresponding secondary structure elements. Fitted residuals are given in Figure S1 and Figure S2, and represent the goodness of the fittings that are graphically displayed in Figure 3 and Figure 10, respectively.

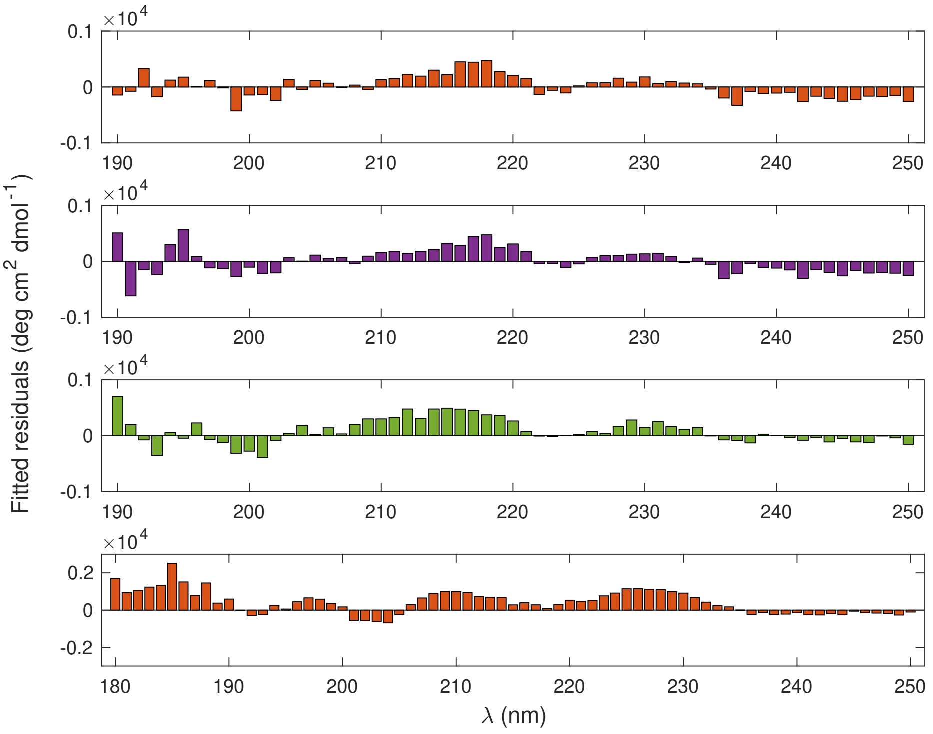

Figure S1. Fitted residuals for spectra recorded in the presence of (from top to bottom) 10 mM NaF (aq.), 150 mM NaF (aq.), 1 mM $ZnCl_2$ (aq.), and TFE (org.). Note that the x- and y-axis ranges vary between the subplots. The fits are graphically presented in Figure 3 in the paper.

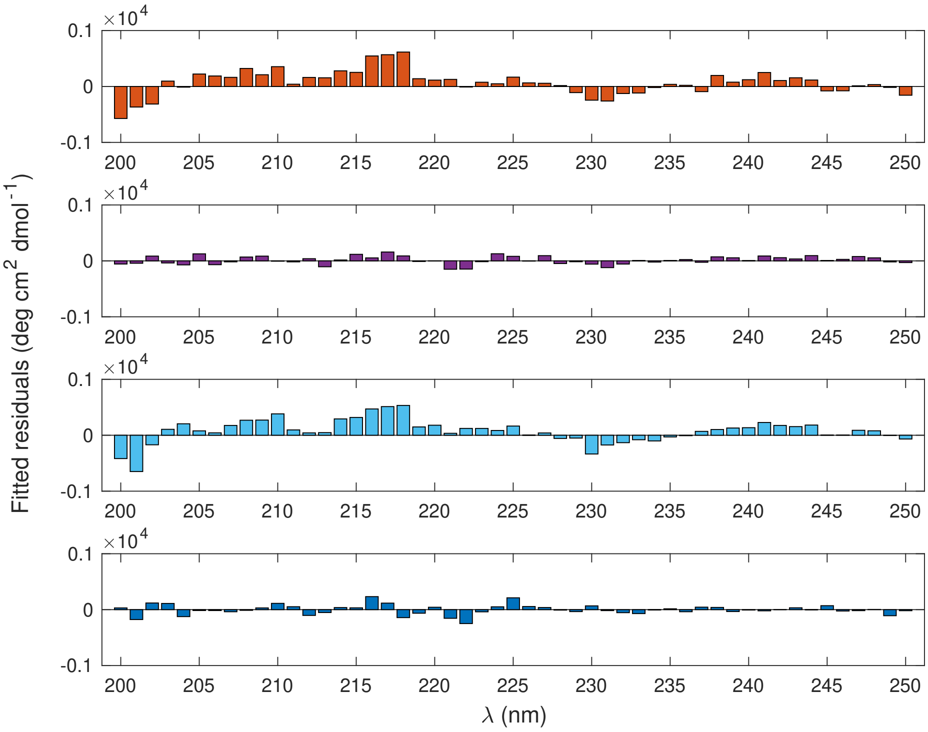

Figure S2. Fitted residuals for spectra recorded in the presence of (from top to bottom) POPC vesicles (aq.), 3:1 POPC:POPS vesicles (aq.), 10 mM NaF (aq.), and TFE (org.). The fits are graphically presented in Figure 10 in the paper.

2. Molecular dynamics simulations

Simulation convergence was assessed by investigating probability distribution functions, auto-correlation functions, and block average error estimates of the radius of gyration and the end-to-end distance, see Figure S3-S6. Although the probability distribution functions in Figure S3 are not identical, they still cover the same region, which is a good indication of a converged system. The auto-correlation functions of the radius of gyration and the end-to-end distance are expected to decrease rapidly and vary around zero for a converged system. In Figure S4 the correlation of all replicates is seen to reach zero within 0.3 µs for the radius of gyration, and within 0.2 µs for the end-to-end distance. Although both properties remain varying around zero, the end-to-end distance seem to be more converged than the radius of gyration. Especially the second replicate (red) seem to have more difficulty of converging. However, when consulting the block average error estimates in Figure S5, the errors are seen to converge to plateau values even for the second replicate. Instead, here the fourth replicate (blue) is shown to be less converged compared to the rest of the replicates. Analysing the concatenated trajectory in Figure S6, gives smooth and well-shaped curves for the probability distribution functions, and both the auto-correlation functions and the block average error estimates look sufficiently converged.

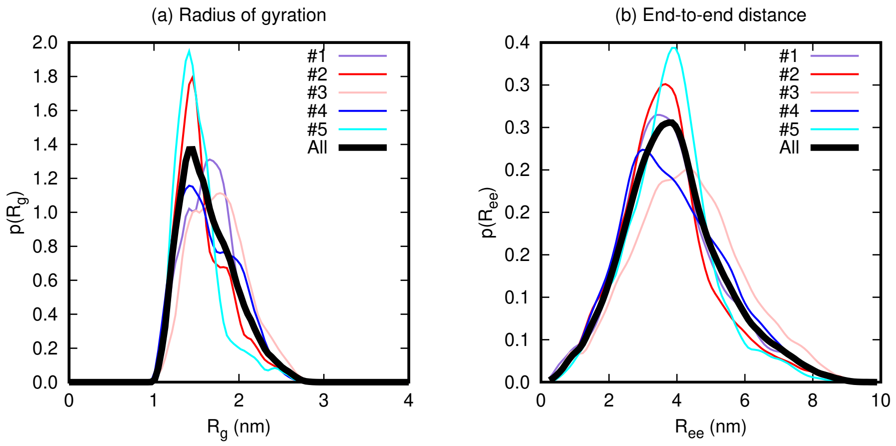

Figure S3. Probability distribution functions of (a) the radius of gyration and (b) the end-to-end distance for each replicate (1-5) and the concatenated trajectory (all).

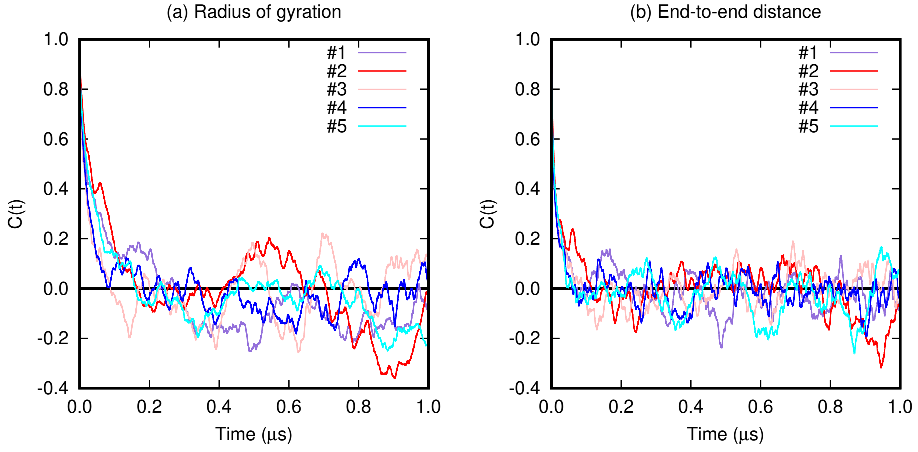

Figure S4. Auto-correlation functions of (a) the radius of gyration and (b) the end-to-end distance for each replicate (1-5).

To further investigate the convergence of the simulation, PCA was performed, and is illustrated for each replicate (1-5) and the concatenated trajectory (all) in Figure S7. Unfortunately, there were fairly large differences between the replicates, so even though they sample the same conformational space in general, different replicates

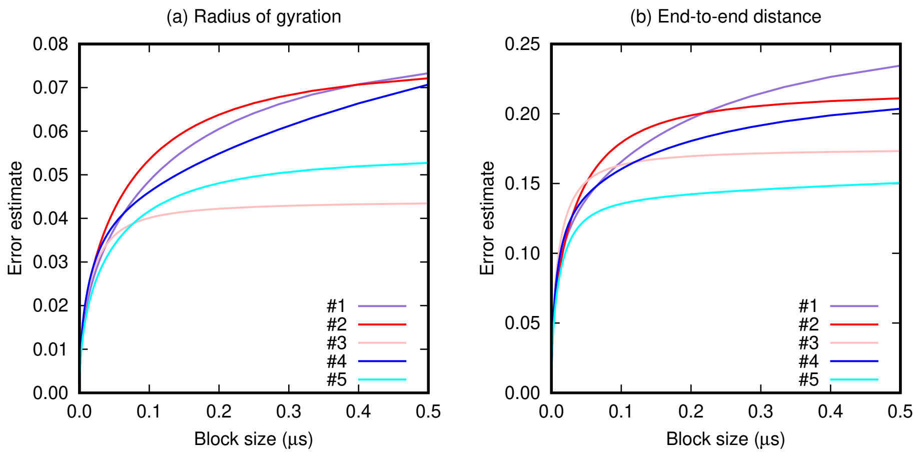

Figure S5. Block average error estimates of (a) the radius of gyration and (b) the end-to-end distance for each replicate (1-5).

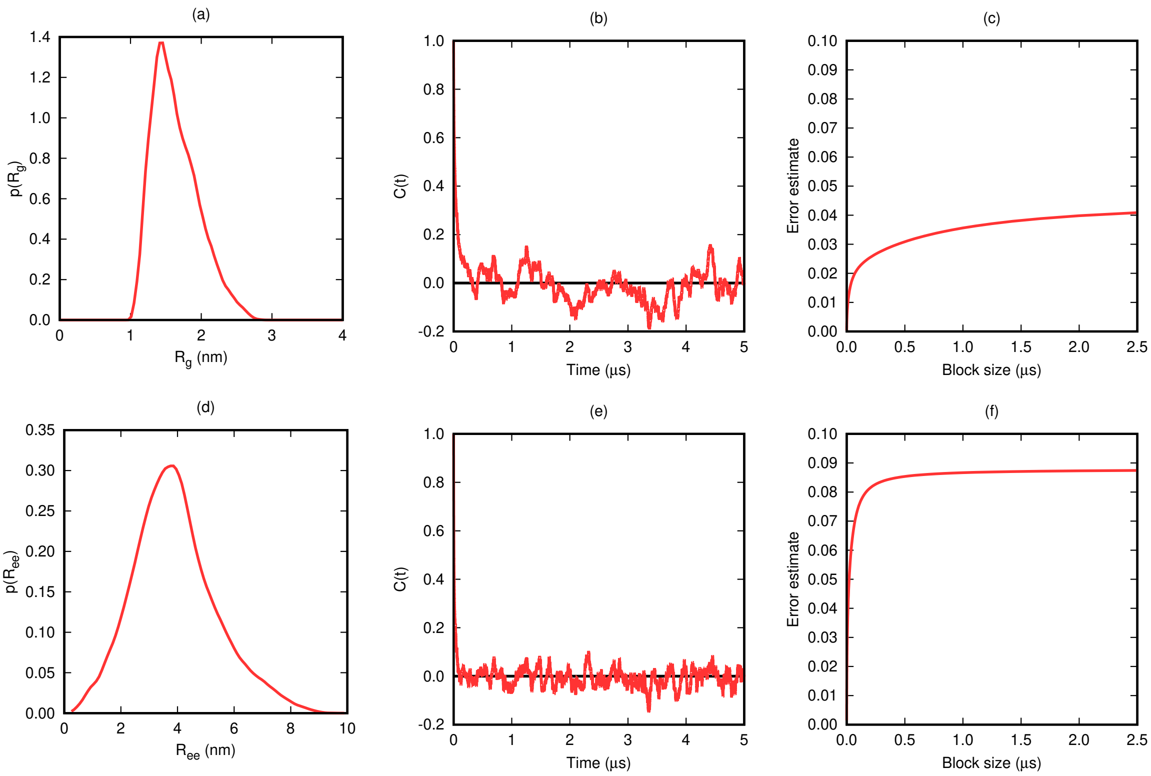

Figure S6. From left to right: probability distribution function, auto-correlation functions, and block average error estimate of (a-c; top row) the radius of gyration and (d-f; bottom row) the end-to-end distance for the concatenated trajectory.

seem to sample different local minima more than others. This is particularly true for replicate 1, 2 and 5. However, PCA of the concatenated trajectory seem to have sampled the entire conformational space sufficiently well.

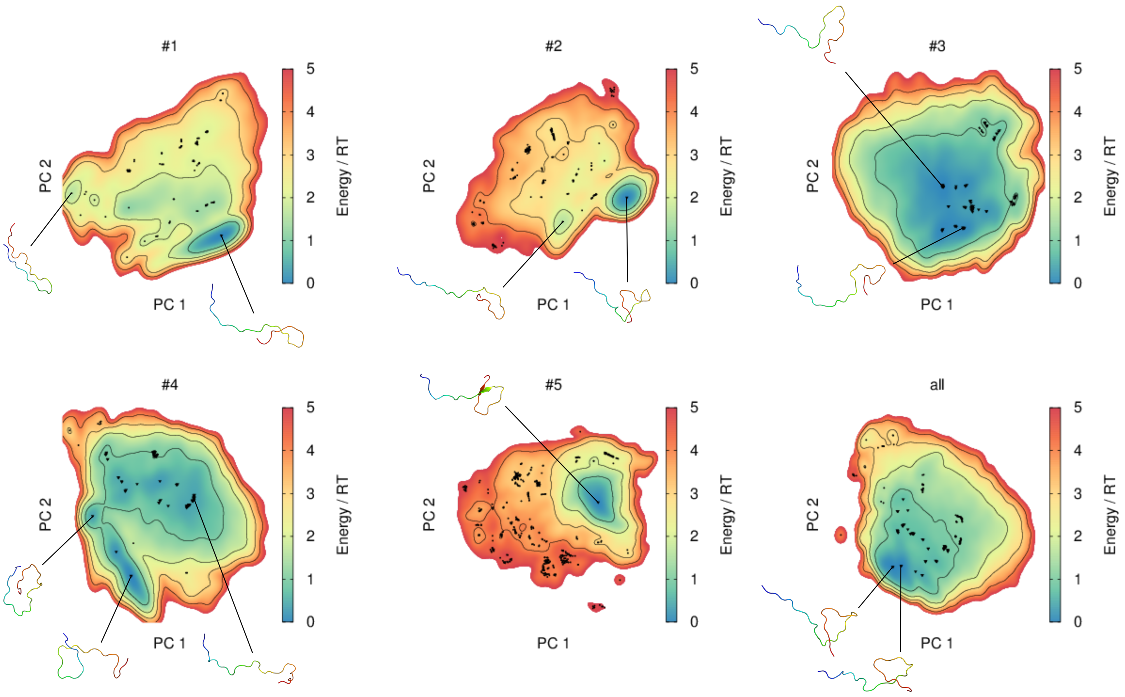

Figure S7. Free energy landscapes of KEIF for each individual replicate (1-5), as well as for the concatenated trajectory (all), using the first two principal components obtained from PCA. Triangles mark energy minima with $RT \leq 1$, and dots mark other local minima with $1 < RT \leq 5$. Snapshots of the representative structure of selected minima are also included.

3. LUVs investigated by dynamic light scattering

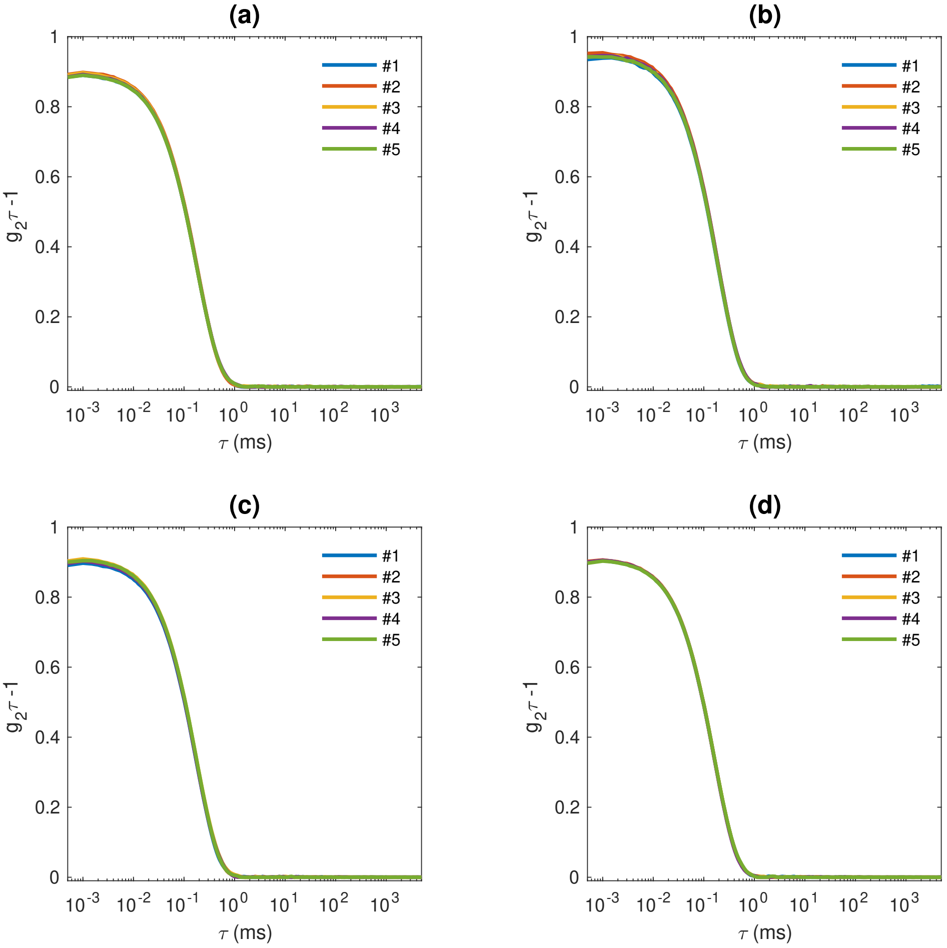

Figure S8. DLS auto-correlation functions obtained with (a) POPS vesicles, (b) POPC vesicles with added KEIF, (c) 3:1 POPC:POPS vesicles, and (d) 3:1 POPC:POPS vesicles with added KEIF. Five consecutive 60 s measurements were performed at scattering angle 173°.

33 ## 4. Additional cryo-TEM images of LUVs

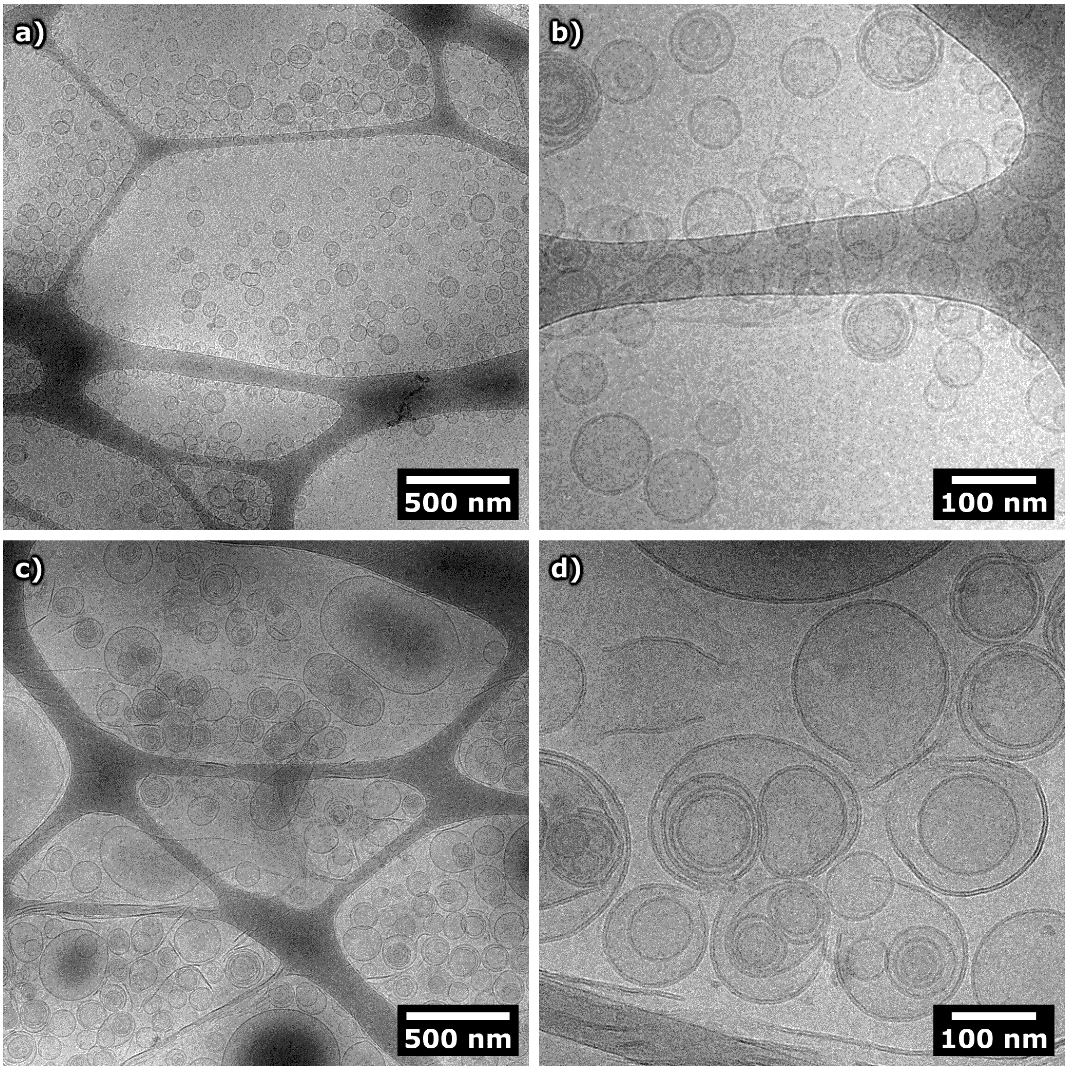

Figure S9. Representative cryo-TEM images of POPC vesicles in the (a, b) absence and (c, d) presence of KEIF, at 10 mM NaF in 20 mM TRIS buffer pH 7.4. The lipid:KEIF molar ratio was 16:1.

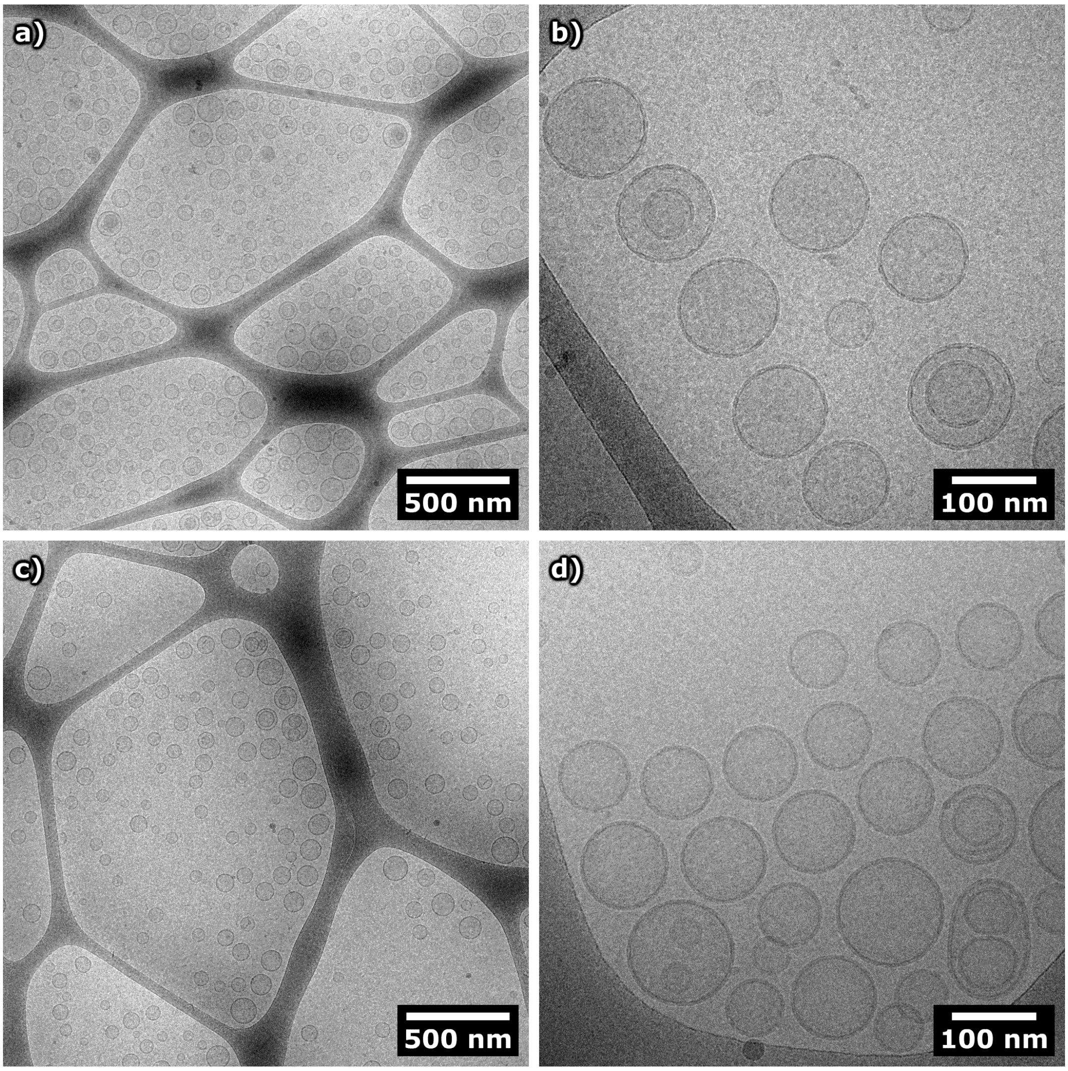

Figure S10. Representative cryo-TEM images of 3:1 POPC:POPS vesicles in the (a, b) absence and (c, d) presence of KEIF, at 10 mM NaF in 20 mM TRIS buffer pH 7.4. The lipid:KEIF molar ratio was 16:1.

5. LUVs' size distributions

Based on the acquired cryo-TEM images, the LUVs' size distributions were obtained by determining their surface area in ImageJ [3], from which their diameter was calculated assuming completely round circles (i.e. spheres in 3D). Considering the small size of the vesicles, flattening effects were assumed negligible; the vast majority of the vesicles were below 150 nm in diameter, and larger (>150 nm) vesicles represented less than 0.5 % of all vesicles analysed. In total, 1822 vesicles were analysed, and the resulting size distributions are displayed in Figure S11. Please, note that the histograms are based on analysing only intact vesicles, and remember that in (b) (i.e. POPC vesicles in the presence of KEIF) the majority of the structures were of irregular shape (incomplete or burst vesicles, discontinuous bilayers, etc.), for which a diameter cannot be defined, and these structures are hence not represented in the corresponding histogram.

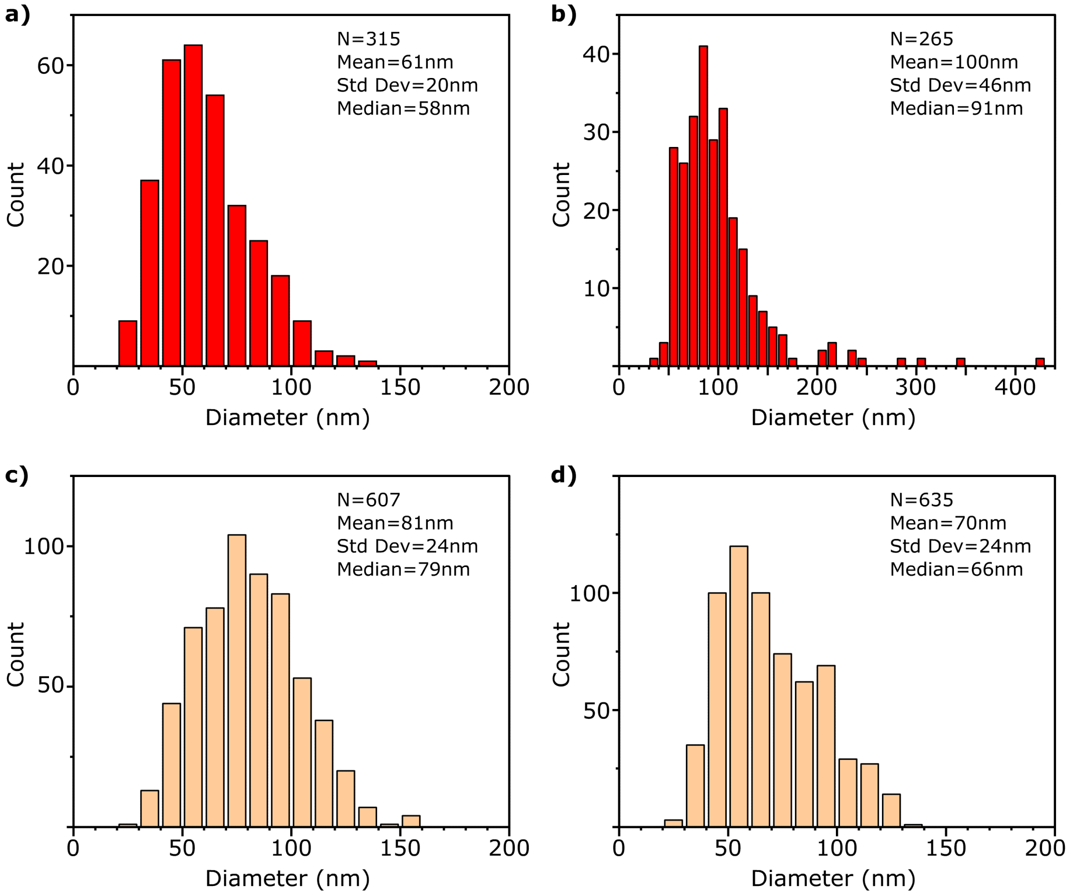

Figure S11. Size distributions, obtained from analysis of cryo-TEM images, of (a, b) POPC and (c, d) 3:1 POPC:POPS vesicles in the (a, c) absence and (b, d) presence of KEIF, at 10 mM NaF in TRIS buffer. The lipid:KEIF molar ratio was 16:1.

References

1. Micsonai, A.; Wien, F.; Kernya, L.; Lee, Y.H.; Goto, Y.; Réfrégiers, M.; Kardos, J. Accurate secondary structure prediction and fold recognition for circular dichroism spectroscopy. *Proc. Natl. Acad. Sci. U. S. A.* **2015**, *112*, E3095–E3103.
2. Micsonai, A.; Wien, F.; Bulyáki, É.; Kun, J.; Moussong, É.; Lee, Y.H.; Goto, Y.; Réfrégiers, M.; Kardos, J. BeStSel: a web server for accurate protein secondary structure prediction and fold recognition from the circular dichroism spectra. *Nucleic Acids Res.* **2018**, *46*, W315–W322.
3. Schneider, C.A.; Rasband, W.S.; Eliceiri, K.W. NIH Image to ImageJ: 25 years of image analysis. *Nat. Methods* **2012**, *9*, 671–675.
